# Kinin Receptor Antagonists as Potential Neuroprotective Agents in Central Nervous System Injury 

**DOI:** 10.3390/molecules15096598

**Published:** 2010-09-20

**Authors:** Emma Thornton, Jenna M Ziebell, Anna V Leonard, Robert Vink

**Affiliations:** 1 Discipline of Anatomy and Pathology, School of Medical Sciences, University of Adelaide, South Australia, Australia; E-Mails: Emma.Thornton@adelaide.edu.au (E.T.); Jenna.Ziebell@adelaide.edu.au (J.M.Z.); Anna.Leonard@adelaide.edu.au (A.V.L.); 2 Adelaide Centre for Neuroscience Research, University of Adelaide, South Australia, Australia

**Keywords:** brain injury, neurotrauma, stroke, neuropeptides, kinins

## Abstract

Injury to the central nervous system initiates complex physiological, cellular and molecular processes that can result in neuronal cell death. Of interest to this review is the activation of the kinin family of neuropeptides, in particular bradykinin and substance P. These neuropeptides are known to have a potent pro-inflammatory role and can initiate neurogenic inflammation resulting in vasodilation, plasma extravasation and the subsequent development of edema. As inflammation and edema play an integral role in the progressive secondary injury that causes neurological deficits, this review critically examines kinin receptor antagonists as a potential neuroprotective intervention for acute brain injury, and more specifically, traumatic brain and spinal cord injury and stroke.

## 1. Introduction

Injuries to the central nervous system (CNS) initiate a complex cascade of secondary biochemical and molecular events that can exacerbate the initial primary injury and the resultant neurological deficits [1]. The primary injury is amenable only to interventions that reduce the risk of insult occurring, whereas the secondary phase of injury can potentially be reduced with pharmacological reagents that target the secondary injury factors. Many factors that contribute to the secondary phase of injury have been identified including blood-brain barrier (BBB) disruption, edema formation, release of neurotoxic excitatory amino acids, oxidative stress and apoptosis, amongst others. The concept of neuroprotection is based on developing appropriate pharmacological interventions that attenuate these secondary processes and improve the functional outcome of patients. The ability of pharmacological agents to limit the biochemical neurotoxicity and secondary cell death has been well established in numerous animal models of traumatic brain injury (TBI), spinal cord injury (SCI) and stroke. However, the results of such neuroprotective strategies have so far produced disappointing outcomes in clinical trials. While the reason for these failures is multifactorial, it is also clear that the targeting of a single factor is of limited benefit when so many different factors contribute to the injury cascade. On the other hand, if a target could be identified that has modulatory effects on multiple injury factors, this target could theoretically provide a multipotential therapeutic approach. One such target is the kinin family of neuropeptides that have been shown to be important modulators of acute CNS injury through multiple mechanisms. 

## 2. Kinins and Their Receptors

All components of the kinin system have been found in abundance throughout both the rat and human CNS, and have attracted interest in neuroprotective research due to their pro-inflammatory action and integral role in initiating neurogenic inflammation following certain types of injury or infection. Of the kinins, bradykinin and substance P (SP) are thought to be the most potent modulators of these injury mechanisms and thus have received the most attention. 

There are two major kinin families: the slow acting bradykinins and the fast acting tachykinins. The bradykinin family includes bradykinin and Lys-bradykinin, also known as kallidin, which are formed by proteolytic cleavage of the protein precursor, kininogen, by plasma and tissue proteases known as kallikreins [2,3]. These kinins bind to the two bradykinin receptors, the constitutively expressed B2 receptor and the B1 receptor whose expression is low in normal conditions but is upregulated following injury, infection and inflammation. In particular, release of cytokines such as interleukin-1β (IL-1β) and tumour necrosis factor-α (TNF-α) are potent modulators of B1 receptor expression [4]. Unlike the B2 receptor, which is activated by both bradykinin and kallidin, the B1 receptor preferentially binds Lys-des-Arg^9^-bradykinin, or bradykinin or kallidin which have had the C-terminal Arg removed by kinases, carboxypeptidase N (in tissue) or carboxypeptidase M (associated with cell membranes) [5]. Once bradykinin has bound to the B2 receptor, the receptor/ligand complex is rapidly internalized before being desensitized and the receptor recycled to the cell surface. In contrast, the B1 receptor and its ligand is slowly internalized and does not undergo desensitization [5]. 

The tachykinin family includes the neuropeptides SP, neurokinin A (NKA) and neurokinin B (NKB), which are located in capsaicin-sensitive neurons, also known as primary sensory neurons, within the CNS, peripheral tissue and non-neuronal cells including endothelial and inflammatory cells [6]. Tachykinins share a common terminal sequence that is essential for their biological activity and thus there is a certain amount of cross reactivity among the tachykinin receptors and their ligands [7,8]. SP has greatest affinity for the tachykinin-1 (NK1) receptor whereas NKA and NKB preferentially bind to the tachykinin-2 (NK2) and tachykinin-3 (NK3) receptor, respectively [9]. Like bradykinin, once SP binds to the NK1 receptor, the receptor and its ligand are rapidly internalized and desensitized [10]. SP expression is most prominent in brain and nerves, although mRNA for other neurokinin receptors is detectable in many cells and regions. However, the predominance of the NK1 receptor in the human adult brain [11] makes SP the main tachykinin of interest in the pathophysiology of CNS injury. 

**Figure 1:**
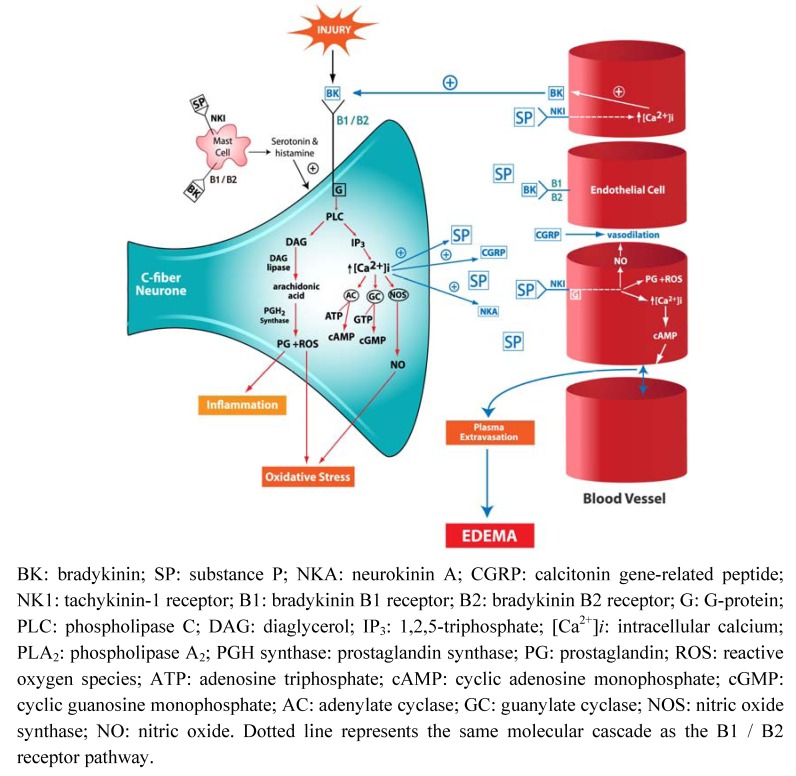
Schematic representation of kinin induced neurogenic inflammation in the central nervous system.

Kinin receptors are found throughout the CNS, where they are expressed on neurons, astrocytes, microglia, endothelial cells and oligodendrocytes [12,13,14,15]. They belong to the family of G-protein coupled receptors and consist of 7 hydrophobic transmembrane domains [4,10]. Activation of kinin receptors stimulates membrane phospholipid metabolism. Once bound to its respective receptor, both bradykinin and SP activates the G-protein coupled to phospholipase C (PLC), which then converts phosphotidylinositol-4-5-biphosphate to 1,2,5-triphosphate (IP_3_) and diaglycerol (DAG) [16,17,18]. This rise in both IP_3_ and DAG occurs within seconds following ligand binding and results in a subsequent rise in intracellular calcium (Ca^2+^). This increase in Ca^2+^ is the key to initiation of many secondary messenger systems in all cells, including the activation of protein kinase C (PKC) and the release of other neurotransmitters (Figure 1). 

Downstream effects of kinin receptor ligand binding also involves the release of arachidonic acid from membrane phospholipids through both a direct activation of phospholipase A_2_ (PLA_2_), as well by the elevated Ca^2+^ induced activation of PLA_2_ and DAG lipase [4]. Arachidonic acid is then metabolized by prostaglandin H (PGH) synthase to prostaglandins and reactive oxygen species (ROS) [19], thereby initiating inflammation and contributing to oxidative stress. In addition, prostaglandins mediate levels of cyclic adenosine monophosphate (cAMP) and cyclic guanosine monophosphate (cGMP), both of which are important in modulating neuronal activity. Although it is not entirely known how prostaglandins do this, it is postulated that they increase cGMP by activating guanylate cyclase [19]. They may also impede vascular autoregulation at sites of tissue injury [20] as increased cGMP levels results in vasodilation and altered ion channel permeability in neurons whereas cAMP along with Ca^2+ ^causes contractions of vascular smooth muscle cells. Indeed, an increase in cAMP has been shown to increase BBB permeability [19]. Furthermore, the NK1 receptor has a 5’ untranslated region containing a cAMP binding protein that responds to elevated levels of cAMP and Ca^2+^ by increasing gene transcription of SP, thereby creating positive feedback for SP [17]. 

The release of nitric oxide (NO) following the binding of kinins to their receptors also contributes to their effects on the vasculature as NO causes both dilation and constriction of vessels. Furthermore NO is involved in BBB dysfunction in CNS injury through its ability to stimulate guanylate cyclase and increase Ca^2+^ [4,18]. Overall, these receptor-signaling pathways reflect the integral role that kinins play in CNS injury.

It is believed that kinins are one of the first mediators of injury mechanisms at the site of the insult [18,21]. Substance P release is initiated by the mechanical stretch of neurons during an injurious event [2] whereas bradykinin is increased after CNS injury through the release of lysosomal enzymes from destroyed cells, which then activate kininogen to release bradykinin or kallidin. Additionally, extravasation of plasma or blood products can initiate Hageman factor XII, which is involved in the coagulation cascade, and is a potent activator of kallikrein [19]. 

Bradykinin also further potentiates the release of SP at the injury site by activating IP_3_ and causing the subsequent rise in intracellular Ca^2+^, a known trigger of neurotransmitter release. However in the terminals of the dorsal root ganglion, bradykinin acts from a distance to release SP by depolarizing the nerve terminal, which opens voltage-sensitive calcium channels resulting in increased intracellular Ca^2+^ and subsequent SP release [19].

The kinins are believed to be involved in most aspects of early neuroinflammation due to the expression of their receptors on most CNS inflammatory cells. Particular focus has been their role in the activation of microglia and astrocytes, which are major mediators of both acute and chronic CNS injury. Resting microglia are vital to the control of normal immune and homeostatic functions within the brain [22]. Normally, these resting microglia are benign to the brain, however once activated through injury or during removal of unwanted cellular debris, they produce inflammatory cytokines, glutamate and quinolinic acid, superoxide (O_2_^-^) radicals and NO [23]. The secretion of NO by microglia is through activation of microglial inducible nitric oxide synthase (iNOS). NO can then react with O_2_^-^ radicals to form perioxynityrite (ONOO), a highly reactive molecule [24]. These ROS and reactive nitrogen species (RNS) contribute to oxidative stress induced CNS injury by causing damage to lipids, proteins and DNA. Both bradykinin and SP activate microglia through their respective receptors, thus contributing to oxidative stress mediated cell death. However, bradykinin has also been shown to play a protective role in the CNS by reducing lipopolysaccharide (Endotoxin: LPS) induced inflammation mediated neuronal death [13].

Activated astrocytes are also involved in CNS injury, however their role in injury is less defined since they secrete protective neurotrophic substances and antioxidant enzymes, as well as the injurious pro-inflammatory cytokines including IL-1β, IL-6 and TNF-α. SP is integral in this proinflammatory role of astrocytes because once activated by SP, nuclear factor-κβ (NF-κβ) translocates to the nucleus to induce cytokine secretion [25,26]. Cytokines such as IL-1β can then increase astrocytic NK1 expression creating a positive feedback between NK1, cytokine release and astrocyte activation [27]. Accordingly, SP has been postulated to be involved in many inflammatory diseases [10]. Importantly for CNS injury, NK1 antagonist treatment has been shown to reduce CNS inflammatory processes [26]. 

Bradykinin has previously been shown to stimulate glutamate release from astrocytes *in vitro* and this release can mediate fast synaptic transmission and synaptic plasticity [28]. However, it can also induce arachidonic acid release [13] and *N*-methyl-D-aspartate (NMDA) receptor mediated rise in neuronal intracellular Ca^2+^ resulting in free radical production, mitochondrial damage and activation of proteases, endonucleases and phospholipases. This deleterious series of events, known as glutamate excitotoxicity, may be a significant contributor to cell death following CNS injury. 

As previously mentioned, the kinins play an integral role in initiating neurogenic inflammation, which is a neurally elicited local painful inflammatory response characterized by vasodilation, protein extravasation and subsequent edema formation [29]. Bradykinin, by binding to its B2 receptor on vascular endothelial cells dilates pre-capillary arteries, increases vascular permeability and causes plasma extravasation [4]. Bradykinin also stimulates nociceptive sensory nerve terminals thus causing the release of neurotransmitters SP, NKA and CGRP [18]. Although both bradykinin and SP are involved in neurogenic inflammation, SP is thought to be the main mediator. Like bradykinin, SP binding to its NK1 receptor on endothelial cells causes opening of post-capillary venular endothelial gaps [30] thus increasing vessel permeability, BBB breakdown and promoting plasma extravasation [6]. CGRP, a most potent vasodilator, increases blood flow, bringing cytokines and inflammatory mediators to the area [31]. However, CGRP can further potentiate SP mediated effects by both increasing the expression of NK1 receptors and also enhancing the bioavailability of SP by competing with SP for catabolism by endopeptidases [32].

In addition, both bradykinin and SP cause degranulation of mast cells resulting in the release of histamine, serotonin and inflammatory mediators, which further increase vascular permeability and plasma extravasation [18,33]. This loss of barrier integrity that occurs in CNS injury also allows peripheral immune cells to cross the once impenetrable barrier to further contribute to or even initiate inflammatory processes within the brain [34]. 

A rapid advance in the knowledge about kinins and their functions was made possible by development of the non-peptide antagonists, which are able cross the BBB to bind to the receptor and inhibit the effects of its ligand. The first non-peptide antagonist was synthesized by Pfizer for the NK1 receptor, and enabled researchers to study the role of SP and NK1 in disease states [10]. To date, Aprepitant, an oral NK1 antagonist is the only NK1 receptor antagonist that has shown potential in a clinical setting, where it is effective in reducing chemotherapy-induced nausea and vomiting [35]. However, human studies in depression and anxiety are ongoing. 

Non-peptide antagonists for bradykinin, in particular for the B2 receptor, were subsequently developed. These bradykinin antagonists greatly improved the knowledge of bradykinin’s role in injury. Previous B1 receptor antagonists had little impact on this field of research due to the induced nature of B1 expression [36]. To date, B2 antagonists have shown therapeutic potential for tumors, by simulating apoptosis in cancer cells, as well as in inflammatory diseases and septic shock [36]. Furthermore, the localization of the B2 receptor within the spinal cord, particularly within areas involved in nociception, suggest an analgesic role for B2 antagonists [37,38,39]. 

Importantly, kinins may play an integral role in the development of CNS injury and therefore their respective receptor antagonists may represent novel neuroprotective therapies. Accordingly, this review will focus on the potential role of kinins and the potential use of their antagonists as a treatment for TBI, SCI and ischemic stroke.

## 3. Traumatic Brain Injury

The injury that occurs after TBI consists of two phases. The initial primary, mechanical damage that occurs at the time of the traumatic incident is irreversible and only amenable to preventative measures designed to either prevent the injury from occurring or minimizing the extent of injury (e.g., seat belts, helmets, airbags, *etc*). The primary damage is followed by a secondary phase, which is a multifactorial process initiated at the time of impact and evolving over the subsequent hours to days [1]. This secondary phase is made up of a variety of physiological, cellular and molecular responses aimed at restoring homeostasis. If not controlled, these processes will lead to secondary injury. Amongst the pathological changes, disruption of the BBB has been demonstrated in the acute post-traumatic period, allowing not only an increase in intracranial pressure (ICP), but also the entry of circulating neutrophils, monocytes and lymphocytes to the injured site, directly impacting on neuronal survival and death (as reviewed in [40]). Increased ICP is thought to be one of the major factors determining mortality and morbidity after TBI.

Over the last 25 years research into therapeutic interventions for brain trauma has evolved from attempts to reduce ICP to attempts to interfere with the underlying pathophysiological mechanisms. Most significantly, recent experimental work has provided evidence for involvement of the kallikrein-kinin system in the progression of edema [41]. In addition, experimental models of CNS injury have determined that bradykinin induces the release of glutamate and aspartate as well as being a potent endothelium-dependant dilator of the brain vasculature [42,43]. Thus, it is not surprising that a significant amount of research has focused on the multifactorial involvement of bradykinin in the secondary phase of TBI. Bradykinin levels were significantly increased at 2 hours following controlled cortical impact before declining over subsequent hours [44]. Moreover, in regards to the mRNA levels of B1 and B2 receptors, expression of the B1 receptor mRNA was increased four-fold at 6 hours after controlled cortical impact injury, although mRNA levels for the B2 receptor did not increase significantly [45]. These results are not surprising given that B1 expression is induced whereas B2 is constitutive expressed. 

Further confirmation for the role of bradykinin and its receptors in TBI has been obtained through the use of genetically engineered mice that are deficient for either bradykinin B1 or B2 receptors. B2 receptor-null animals had 50% less brain water content following injury than wild-type animals and performed better on functional outcome tests [44]. In contrast, another study reported that B2 receptor-null mice had an improved neurological outcome but no difference in brain edema, although it may be possible that up-regulation of another compensatory pathway may have occurred in response to the B2 receptor being genetically absent. In addition, both B2 receptor-null animals and animals treated with a B2 receptor antagonist had decreased accumulation of neutrophils 24 hours post-TBI as well as reduced iNOS mRNA levels. Furthermore, contusions in B2 receptor-null mice were significantly smaller than wild-type and B1 receptor-null animals [46]. The authors concluded that detrimental effects of bradykinin after TBI are mainly mediated through B2 receptors on cerebral vessels, the cerebrovascular effect, driving the formation of brain edema, vasodilation, and/or changes in cerebral blood flow. 

Nonetheless, a role for B1 receptors in the genesis of edema has also been recently suggested. In a murine model of experimental cryolesion, B1 receptor-null animals were reported to have smaller lesions with reduced BBB dysfunction and edema formation [47]. Additionally, the therapeutic treatment of wild-type mice with the B1 receptor inhibitor, R-715, mirrored the results obtained from the knockout studies. The authors failed to find similar neuroprotection when the B2 receptor was knocked out or inhibited therapeutically. This study is contradictory to much of the previous literature, and the authors themselves conclude that the contradictions may be due to the different model which they employed to study this phenomenon. In any case, these collective observations suggest a detrimental role for bradykinin receptors in TBI and provide compelling evidence that therapeutic inhibition of these receptors may be beneficial for outcome of patients following TBI.

The first experimental TBI studies utilizing a B2 receptor antagonist used aprotinin, a non-specific kallikrein inhibitor, to therapeutically target the vasogenic edema produced from cortical cold lesion in rabbits [48]. However, prior to the development of a non-peptide bradykinin antagonist, these compounds were plagued with low potency and poor *in vivo* stability [49]. The first non-peptide bradykinin B2 receptor antagonist, CP-0127, revealed no significant toxicity in pre-clinical animal toxicity or phase I human volunteer studies [49]. Anatibant^®^ (LF 16-0687 Ms), another non-peptide B2 receptor antagonist, has also been trialed in experimental TBI. Rats subject to closed head injury had reduced brain edema at 24 hours as well as improved neurological function at day 3 and 7 following Anatibant^®^ infusion administered from 1 to 24 hours post-injury [50]. Furthermore, experimental rat models of both cryolesion and controlled cortical impact have shown Anatibant^®^ to reduce brain edema [41,51]. Along with this reduction in brain edema, Anatibant^®^ treatment also reduced ATP breakdown products hypoxanthine and xanthine [41]. These products are formed following injury, especially in times of limited oxygen, and contribute to the production of potentially harmful free radicals. In addition, administration of Anatibant^®^ 30 minutes after diffuse injury reduced neurological deficit by 26% and brain edema by 22% [46]. In agreement with Hellal and colleagues [46], Ongali *et al*. [45] reported that subcutaneous treatment with Anatibant^®^ 30 minutes post-injury significantly reduced BBB permeability at 4 hours. However Anatibant^®^ may need to be administered almost immediately after injury, as after cryolesion injury there was no effect if given 30 to 60 minutes post-injury [52]. Nonetheless when interpreting these results, one must take into account the different types of brain injury employed in these studies. 

Recently studies have utilized autoradiographic technology, not only to determine the brain regions where B1 and B2 receptors are located normally, but also to determine the displacement of radioligands following treatment with the receptor antagonist Anatibant^®^ [45]. This study showed the ability of Anatibant^®^ to cross the normal BBB and displace the B2 receptor radioligand from various regions including the forebrain, basal ganglia and hindbrain. This displacement was present in all areas at 1 hour and persisted for up to 4 hours after the injection. Following disruption of the BBB by injury, Anatibant^®^ is able to penetrate the brain more readily, which may account for its neuro- and vascular protective effects [45].

Following on from the promising experimental work, a single-dose, three-arm, placebo-controlled, phase I clinical study of Anatibant^®^ in patients with severe TBI was published in 2005. During this trial Anatibant^®^ was administered as a subcutaneous injection at either 3.75 mg or 22.5 mg. The authors reported that the drug was well tolerated in severe TBI patients with no unexpected adverse outcomes, with the recommendation to begin phase II trials to determine a dose-response [53]. Unfortunately, the phase II placebo controlled trial was abandoned after collecting data from just over half the number of patients in the planned sample size [54]. Of 163 patients treated with Anatibant^®^, 43 were reported to have adverse events within 14 days of treatment compared to 11 of the 57 placebo patients. A response letter was written by the drug company involved suggesting the study conditions were flawed with inadequate guidelines given to investigators [55]. In wake of this latest trial there is a cloud over whether Anatibant^®^ has beneficial or detrimental effects on TBI patients. 

Other clinical trials utilizing bradykinin antagonists have been more successful. In a randomized, single-blinded study in head injured patients, CP-0127 performed well [54]. CP-0127 was reported to prevent the pathological rise in ICP and the subsequent neurological deterioration, thereby reducing the need for surgical intervention or manipulation of ICP. However, the trial was conducted on a highly selective group of patients with focal lesions. Another more robust phase II prospective, randomized, double-blinded clinical study was presented in 1999 where Bradycor™ (deltibant, CP-1027) or placebo was administered as a continuous 5-day infusion, beginning within 12 hours of injury [56]. Patients were followed for the first 14 days of their hospital stay and then followed up at 3 and 6 months post-injury. Bradycor™ was well tolerated with no adverse events attributable to the compound. Importantly, there were positive trends reported for ICP, therapy intensity level, neuropsychological tests both during the initial stay and at the 3- and 6- month follow ups [55]. Whilst these clinical trials provide encouraging evidence that bradykinin receptor antagonists may potentially afford neuroprotection in TBI, it should be noted that safety and efficacy of B2 receptor antagonists have not been reliably ascertained and more work needs to be undertaken using well organized, randomized clinical trials [57].

When considering mechanisms of action, one should not dismiss the fact that bradykinin potentiates the release of the tachykinin SP, which has been recently implicated as a critical factor in the formation of post-traumatic edema [29,58,59]. Statistically higher levels of SP have been detected in the blood 30 minutes port-injury before declining back to sham levels by 5 hours [58]. It is thought that this SP is released in the vicinity of the vasculature by mechanical stretch of the perivascular neurons before entering the bloodstream [60]. This perivascular release has been postulated to be associated with the early, but transient, opening of the BBB and the subsequent formation of vasogenic edema. Notably, both bradykinin and SP can be modulated by angiotensin-converting enzyme (ACE), through its involvement in their degradation. ACE breaks down hydrolytic bonds in such a way that kinins are unable to bind to their receptor [61]. Indeed, recently it was shown that treatment with the ACE inhibitor Captopril further increased SP immunoreactivity in injured brain at 5 hours after TBI and resulted in significantly worsened outcome [62]. To ensure this was a class effect a second ACE inhibitor, Enalapril, was also administered and shown to produce similar effects. 

The role of SP in post-traumatic vasogenic edema formation has recently been studied utilizing diffusion weighted magnetic resonance (MR) imaging [63]. These studies reported that in a model of diffuse axonal injury in rats, TBI increased the apparent diffusion coefficient reflecting the increased diffusion distance of water related to vasogenic edema. Notably, when animals were pre-treated with capsaicin, a compound that depletes neuropeptides including SP, the apparent diffusion coefficient was lowered [63]. Accordingly, capsaicin treatment resulted in a profound reduction in edema at 5 hours post-injury as well as reduced BBB permeability and improved motor and cognitive outcome. Furthermore, another set of studies using magnetic resonance spectroscopy demonstrated that administration of an NK1 antagonist increased brain free magnesium concentration [64], which has previously been shown to be strongly associated with outcome after TBI [65]. The NK1 antagonist also reduced edema as measured by MR imaging plus wet weight/dry weight analysis, reduced BBB permeability and improved functional outcome [58,66]. The data therefore confirm that the release of neuropeptides in the form of neurogenic inflammation play a critical role in the secondary events associated with TBI.

## 4. Spinal Cord Injury

SCI, like TBI, involves both primary and secondary mechanisms of injury. Of particular interest is blood spinal cord barrier (BSCB) disruption, which is associated with the development of profound post-traumatic edema that leads to functional deficits. The BSCB exists at the capillary level and regulates the molecules that can enter the spinal cord tissue. Electron microscopy has demonstrated that in the BSCB the endothelial cells connected by tight junctions are surrounded by a think basement membrane, as observed in the BBB of the brain [67]. Thus, the BSCB resembles the BBB in many ways, not only in function but also structurally [67,68,69]. It has been well established that BSCB disruption [70,71] and edema formation [69,72,73,74,75,76] occur following SCI. Furthermore, such processes have been associated with a worsening of function, whilst restoration of the BSCB improved functional outcome [77,78]. Thus the development of pharmacological intervention that reduce BSCB disruption is of utmost importance. 

As mentioned earlier, the kallikrein-kinin system has been implicated in posttraumatic vascular injury of the CNS and in vasogenic edema. In the spinal cord, kininogen and its conversion to vasoactive kinins significantly increased following a weight drop model of SCI in rats [79]. Therefore it is not surprising that the kallikrein-kinin system has also been targeted for the development pharmacological intervention following SCI. Like in TBI, bradykinin has been implicated in the disruption of the BSCB observed following SCI. Furthermore, pre-treatment with a B2 receptor antagonist (B9430) significantly reduced BSCB disruption immediately following a bilateral compression model of SCI [80]. Similarly, Sharma and colleagues found that pre-treatment with the B2 receptor antagonist HOE-140 significantly reduced BSCB permeability following a focal model of SCI [81]. These investigations clearly demonstrate that bradykinin is involved in the disruption of the BSCB following trauma. However, two phases of BSCB disruption have been observed, one immediately following SCI and the second at 72 hours post-injury [80]. Whilst the B9430 was able to attenuate BSCB disruption immediately following injury, there was no significant difference at the 72 hour time point. It is well known that bradykinin has a short half-life [82] and that its concentration begins to fall quite quickly following an initial tissue insult [19]. This suggests that other mediators may play more important roles at these later time points. 

One candidate for this later disruption of the BSCB is SP, although at present, no such research has investigated the role of SP in neurogenic inflammation following SCI. However, SCI causes changes in SP expression within the spinal cord. Following a focal model of SCI a significant increase in the content of SP was observed at both 1 and 2 hours [76]. This result was detected not only in the injured spinal segment but also in the samples removed 5 mm proximal and distal to the lesion [83]. In contrast, a decrease in SP has also been observed at both 5 hours [83] and later at 7 days post-SCI [84]. In a transection model of SCI, the level of SP immunoreactivity was increased at 5 days, 2, 5, 8 and 12 weeks post injury [85]. The differences in SP expression following injury may simply be due to the different injury models employed, although further investigation is required to clarify the issue.

In addition to alterations in SP, concurrent changes in NK1 receptor expression have also been noted. At 1 week following a thoracic spinal cord transection, a significant increase of NK1 below the injury site within the dorsal horn and lamina X was observed [86]. This increase may correlate with the previously demonstrated increased SP within these regions following a transection injury [85]. Vita and colleagues further demonstrated an increase of NK1 receptors within the ventral horn above the injury site, which again correlates with previously reported SP increases [87]. Furthermore, at the cervical level a significant increase of NK1 density was also observed at 1 week post-injury within some lamina of the dorsal horn. This increase in receptor density has not been matched by previous studies, where no increase or even a decrease in SP content has been observed [85].

Further confirmation for a role of SP in BSCB dysfunction and genesis of edema following SCI has been indirectly obtained by examining the effects of naloxone [76,84]. Administration of naloxone, an opiate antagonist, has been shown to significantly reduce the development of edema [76], increase blood flow [88], reduce ischemia [89] and improve neurological function [84]. Naloxone treatment was most effective when it inhibited the kappa-opioid receptor [76,90]. Administration of a kappa-opioid antagonist has also been demonstrated to improve neurological outcome following TBI [91]. It should be highlighted that stimulation of the kappa-opioid receptor facilitates the release of SP [92]. Accordingly, inhibition of kappa-opioid receptors can lead to a reduction in SP release [93], which in part, may account for the beneficial effects of naloxone and other kappa-opioid receptor antagonists in SCI.

Finally, of particular interest is the fact that the changes in SP expression are observed at later time points, supporting the proposition that SP may play an important role in mediating injury at later time points, or indeed both at early and late time points. Thus, it may be that whilst bradykinin is involved in the early development of neurogenic inflammation, such processes are then potentiated through bradykinin mediated release of SP. 

## 5. Ischemic Stroke

Ischemic stroke results from a disturbance of the blood supply to the brain caused by a blockage due to either thrombosis or an embolism. This reduction or loss of blood flow results in cerebral tissue becoming hypoxic, and the initiation of secondary injury mechanisms that over time, cause neuronal cell death and neurological deficits. Thus restoration of blood flow to the brain is of upmost importance. To date, the only clinical treatment available for ischemic stroke is the intravenous administration of tissue-type plasminogen activator (tPA), which breaks down clots to restore normal blood flow. Unfortunately, not only does the restoration of blood flow also initiate reperfusion related injury mechanisms, tPA must be administered within 4.5 hours of symptom onset [94]. Thus the search for novel neuroprotective agents that may reduce both hypoxic and reperfusion injury continues.

Bradykinin and the tissue kallikrein-kinin system have been implicated in ischemic injury both in the periphery and in the CNS, where it plays a critical role in cell death by potentiating neuroinflammation, vasodilation and increasing vascular permeability resulting in edema. In ischemic stroke both kallidin and bradykinin expression are increased early. In experimental models of ischemic stroke bradykinin levels were elevated during the first 24 hours following reperfusion with maximal expression at 12 hours [95]. However, bradykinin returns to normal levels by 24 hours, suggesting that the rise occurs early but is not sustained. In contrast, in human ischemic stroke bradykinin levels were similar to that of controls, although plasma kallidin levels were increased at 24 hours and remained elevated for the subsequent week [96]. However the earliest time point studied was 24 hours, and therefore the rise in bradykinin may have been missed. These results suggest a role for the tissue-kallidin system in potentiating injury during ischemia/reperfusion. Indeed in animal models of cerebral ischemia, tissue bradykinin levels show a marked correlation with tissue injury and brain swelling [97]. 

Along with the increase in bradykinin and kallidin levels, there is a concurrent increase in bradykinin receptors B1 and B2. This increase in receptor expression has been observed as early as 4 hours and was still apparent at 24 hours following reperfusion [95]. Interestingly, the receptors showed different patterns of expression, with B2 receptors primarily found on neurons, whereas B1 receptors were expressed on astrocytes in the ischemic penumbra. Moreover, in a murine model of ischemic stroke, the expression of B1 receptor mRNA was substantially greater than B2 receptor mRNA expression at 24 hours post-injury [98]. This differential pattern of expression suggests that these receptors modulate different aspects of ischemic injury [99], and potentially both B1 and B2 receptor antagonists may provide protection in ischemic stroke. 

The predominant expression of B2 receptors on neurons has led much of the research on neuroprotection in ischemic stroke to focus on antagonism of the B2 receptor, which has provided substantial protection in animal models of ischemic stroke. Continuous or multiple systemic administration of the B2 antagonists CP-0597 and LF 16-0687 Ms, following transient middle cerebral artery occlusion (MCAO) reduced edema and infarct size and improved behavioral deficits [21,100]. Notably, low doses of LF 16-087 demonstrated greater neuroprotection than higher doses, which the authors postulated could be due to depression of cholinergic transmission or increase in B1 receptor expression due to prolonged blocking of B2 receptors [21]. However, it was recently shown that the long acting B2 antagonist, bradyzide, reduced the expression of B1 receptors [99], which argues against long term B2 inhibition increasing the effects of the B1 mediated injury pathway. This study also demonstrated that bradyzide, had greater neuroprotective efficacy than the B1 receptor antagonist, SSR240612, as it maintained BBB integrity by protecting endothelial cells and tight junctions. Along with protection of the BBB, bradyzide reduced the release of pro-inflammatory cytokines and improved neurological outcome [99]. Further confirmation that the B2 receptor plays a critical role in ischemic injury is that B2 receptor knockout mice had reduced infarct size and brain water content, resulting in an improvement in function [95]. 

In contrast to these studies, Austinat and colleagues showed that the absence or antagonism of the B1 receptor provided greater neuroprotection than inhibition of B2 receptors in ischemic injury [98]. In this study, B1 receptor-null mice that underwent transient MCAO had approximately a 30% reduction in infarct size. This was deemed to be functionally relevant as B1 receptor-null mice had improved neurological and motor outcome compared to wild type mice, and in addition had reduced edema formation and IL-1β production at 24 hours. The role of B1 receptors in ischemic injury was confirmed by treating wild type mice with the B1 receptor antagonist, R-715, which produced similar protection and improvement in function to the B1 receptor-null mice. In contrast to the above, B2 receptor-null mice and treatment with the B2 antagonist, Hoe-140, did not afford protection in ischemia, suggesting that ischemic injury was mainly mediated by B1 receptors. Interestingly, bradyzide, which provided greater protection than the B1 receptor antagonist, SSR240612, also reduced the expression of B1 receptors [99]. Thus the greater efficacy produced by this antagonist may be due to its ability to reduce both B1 and B2 mediated injury cascades. These results confirm that bradykinin plays an integral role in early ischemic injury and that both B1 and B2 receptor antagonists may represent novel neuroprotective therapies in ischemic stroke.

Despite the early detrimental effects of bradykinin and their receptors in ischemic stroke, treatment with bradykinin antagonists may still be beneficial for longer-term neuronal survival through ischemic post-conditioning. Ischemic post-conditioning is a relatively new phenomenon and is defined as a “sublethal stimuli or insult performed immediately or up to 2 days after cerebral ischemia” which affords neuroprotection to vulnerable neurons [101]. The mechanisms by which post-conditioning protect neurons remains relatively unknown. However original studies used interruptions to reperfusion to induce post-conditioning, implying that changes in cerebral blood flow and consequent changes in BBB integrity and inflammation are involved (for review, see [101]). Bradykinin plays an integral role in cerebral blood flow, barrier function and inflammation and therefore bradykinin in addition to other modes of post-conditioning such as treatment with norepinephrine or 3-nitropropionic acid or another short ischemic insult, are neuroprotective in ischemic stroke [102,103].

Indeed, gene transfer treatment using the adenovirus carrying the human tissue Kallikrein gene promoted cell survival, angiogenesis, neurogenesis and gliosis in the penumbra both immediately [104] and at 8 hours following ischemia/reperfusion injury [105]. Furthermore, bradykinin by binding to both B1 and B2 receptors causes the release of NO [106]. The production of NO during the later stages of stroke may be beneficial to neurons as it can scavenge superoxide radicals, and promote proliferation of both neurons and endothelial cells [105]. Further post-conditioning studies have shown that treatment with bradykinin as late as 2 days following the initial ischemic insult provided neuroprotection for the particularly vulnerable CA1 hippocampal neurons. Post-conditioning by bradykinin regulated antioxidant enzymes expression, superoxide dismutase and catalase, and protected mitochondria by reducing oxidative stress and subsequent cell death [107]. It has been suggested that the B2 receptor may play an integral role in this protective effect of bradykinin as B2 receptor-null mice had larger infarct sizes, and greater neurological deficits and mortality [108]. However, the validity of this study has been questioned [109] and no other studies have reported that a lack of B2 receptor is detrimental in ischemic stroke.

Bradykinin has also been shown to possess anti-inflammatory properties as it inhibited LPS induced TNF-α and IL-1β release from microglia *in vitro* [13]*. *The authors postulated that bradykinin modulated microglial function by increasing prostaglandin synthesis, resulting in greater microglial cAMP production and a negative feedback for cytokine production. Overall however, bradykinin may be beneficial following ischemic stroke if administered at the later stages, while in the early stages it is particularly detrimental as it induces both an inflammatory response and neurogenic inflammation. Furthermore, its ability to increase SP release is likely to further contribute to ischemic injury in a similar mechanism as is seen in TBI and SCI. 

Despite the detrimental role of SP in CNS injury, little research has been undertaken to date on the role of SP in ischemic stroke. It is known that SP release is increased during an ischemic insult in both animal models and clinical stroke. In transient MCAO, SP expression was increased at 24 hours post-reperfusion, with this increase particularly apparent in perivascular, neuronal and glial tissue within the penumbra [110]. As previously discussed SP, like bradykinin, initiates neurogenic inflammation resulting in increased vascular permeability, plasma extravasation and subsequent genesis of edema. Accordingly, the rise in SP expression was associated with edema formation [110]. Interestingly in the clinic, transient ischemic attacks produced a greater increase in serum SP levels in than in complete stroke, although both transient and complete stroke resulted in higher serum SP levels than control patients [111]. These results suggest that SP is an important modulator of ischemic injury and may be particularly integral to reperfusion injury, which is known to contribute to neuronal cell death. During a stroke, blood flow is limited for a period of time causing cerebral tissue to become hypoxic. In an *in vitro* model of hypoxia, SP release was increased in the carotid body, in a Ca^2+^ dependent manner [112]. The carotid bodies are the major peripheral chemoreceptors, which detect changes in partial pressure of oxygen in arterial blood, then relay signals to the brain stem to instigate the required changes to improve oxygenation to the brain. Thus carotid bodies and SP may be integrally involved in ischemic stroke where there is both hypoxia and reperfusion injury. Therefore inhibiting SP effects may be beneficial. 

Indeed, intracerebral ventricular administration of the potent NK1 antagonist, SR140333, significantly reduced infarct volume and improved functional deficits at 24 hours following focal ischemia in the rat [113]. Similar findings have also been reported in a transient model of focal ischemia in rats where intravenous administration of an NK1 antagonist was shown to reduce edema formation and significantly improve functional outcome [114]. In addition, in myocardial ischemia, treatment with the NK1 antagonist L-703,606 reduced post-reperfusion ischemic injury in the hearts of magnesium deficient rats [115]. A magnesium deficient diet in these animals is known to induce neurogenic inflammation with associated increases in SP and proinflammatory cytokines [116]. These results suggest that administration of a NK1 antagonist during ischemic injury may provide a novel avenue for neuroprotection following ischemic stroke. 

## 6. Conclusions

Kinins, and particularly bradykinin and SP, play an integral role in the injury processes that occur following TBI, SCI and ischemic stroke. This review has focused on their ability to induce neurogenic inflammation resulting in vasodilation, plasma extravasation and genesis of edema, while indicating that they have modulatory effects on a number of other secondary injury factors include excitotoxicity, oxidative stress, and ion gradients. Moreover, kinins are potent pro-inflammatory mediators by binding to their respective receptors located on all CNS inflammatory cells. Both neuroinflammation and edema are integral to the progressive secondary injury mechanisms that lead to the development of neurological deficits. Thus kinin receptor antagonists may represent novel neuroprotective agents for the treatment of CNS injury.
